# Disorder Predictors Also Predict Backbone Dynamics for a Family of Disordered Proteins

**DOI:** 10.1371/journal.pone.0029207

**Published:** 2011-12-15

**Authors:** Gary W. Daughdrill, Wade M. Borcherds, Hongwei Wu

**Affiliations:** 1 Department of Cell Biology, Microbiology and Molecular Biology and Center for Drug Discovery and Innovation, University of South Florida, Tampa, Florida, United States of America; 2 Department of Cell Biology, Microbiology and Molecular Biology, University of South Florida, Tampa, Florida, United States of America; 3 Center for Drug Discovery and Innovation, University of South Florida, Tampa, Florida, United States of America; University of South Florida College of Medicine, United States of America

## Abstract

Several algorithms have been developed that use amino acid sequences to predict whether or not a protein or a region of a protein is disordered. These algorithms make accurate predictions for disordered regions that are 30 amino acids or longer, but it is unclear whether the predictions can be directly related to the backbone dynamics of individual amino acid residues. The nuclear Overhauser effect between the amide nitrogen and hydrogen (NHNOE) provides an unambiguous measure of backbone dynamics at single residue resolution and is an excellent tool for characterizing the dynamic behavior of disordered proteins. In this report, we show that the NHNOE values for several members of a family of disordered proteins are highly correlated with the output from three popular algorithms used to predict disordered regions from amino acid sequence. This is the first test between an experimental measure of residue specific backbone dynamics and disorder predictions. The results suggest that some disorder predictors can accurately estimate the backbone dynamics of individual amino acids in a long disordered region.

## Introduction

Intrinsically disordered proteins (IDPs) are widespread in eukaryotes and overrepresented in a number of human diseases, including cancer and neurodegenerative diseases [1], [2], [3], [4], [5], [6], [7], [8], [9], [10], [11], [12], [13], [14], [15]. While the development of robust generalizations to describe the structure and function of IDPs is ongoing, a number of algorithms have been developed to identify IDPs and distinguish them from ordered proteins. This is achieved using the compositional differences in the amino acid sequences observed between ordered and disordered proteins [16], [17], [18], [19]. These algorithms can identify disordered regions that are 30 amino acids or longer with 75–80% accuracy, but it is unclear whether they provide any information about backbone dynamics at the level of single amino acid residues.

Three popular disorder predictors, VL-XT (www.pondr.com), IUPred (iupred.enzim.hu), and VSL2B (ist.temple.edu) were selected to investigate whether there is a relationship between disorder probability and backbone dynamics. These algorithms were tested because they predict disorder using different principles. The VL-XT predictor integrates three feed forward neural network predictors (NNP). One NNP was trained using 8 long disordered regions identified from missing electron density in x-ray crystallographic studies, and 7 long disordered regions characterized by nuclear magnetic resonance (NMR) spectroscopy [19]. The other two NNPs were also trained using missing electron density from x-ray crystallographic data [20]. For these NNPs, N- and C-terminal disordered regions of 5 or more amino acids were used in the training set. The abbreviation, VL-XT, stands for the use of *V*arious methods to characterize *L*ong disordered regions combined with *T*erminal disordered regions that were characterized using *X*-ray crystallography. When making predictions, VL-XT gives an output between 0 and 1 that is smoothed over a sliding window of 9 amino acids. If a residue value exceeds or matches a threshold of 0.5 the residue is considered disordered. IUPred is an abbreviation for *I*ntrinsically *U*nstructured protein *Pred*ictor. This algorithm distinguishes ordered regions from disordered regions by estimating pairwise interaction energies. This interaction energy is determined using amino acid composition, the local sequence environment, and potential intramolecular interaction partners. When predicting long disordered regions, IUPred calculates interaction energies over a 100 residue sequential neighborhood. IUPred also provides an output that varies between 0 and 1 with a threshold for the transition between order and disorder of 0.5 [21], [22]. VSL2B was also considered, it is a disorder prediction algorithm developed by the same group that developed VL-XT [23], [24]. VSL2B stands for *V*arious *S*hort *L*ong predictor and is the second version of VSL, and B denotes that it does not include the PSI-BLAST feature set. VSL2B was developed using a larger database of experimentally characterized IDPs than VL-XT, it incorporates 26 sequence-based features, including secondary structure prediction. VSL2B is more accurate than VL-XT at predicting short disordered regions; it accomplishes this by having a two tiered prediction method with separate prediction mechanisms for long or short disordered regions that are then combined by a Meta predictor [25], [26]. VSL2B also provides an output scale from 0 to 1 with 1 being most disordered with a disorder threshold of 0.5. All of these predictors do a good job making a gross distinction between the ordered and disordered regions in proteins, but it remains unclear if the variation observed in the prediction values has any relationship to the backbone dynamics of individual amino acids.

NMR spectroscopy is the best available tool for investigating the molecular motions of IDPs [27], [28], [29], [30]. In particular, the NHNOE provides an unambiguous measure of protein backbone motion at single residue resolution. The value of the NHNOE is proportional to the local correlation time for rotational motion. As expected, local rotational correlation times for IDPs are typically shorter than those observed for ordered proteins with a similar molecular weight and can range from 100 ps to 1 ns. IDPs also have more variation in their local correlation times than ordered proteins and this variation reflects the formation of transient secondary structure and, in some cases, transient long-range contacts.

The transactivation domain of the tumor suppressor p53 (p53TAD), is being used as a model for investigating the structure and dynamics of disordered proteins. There is a wealth of functional data for this domain and several NMR studies have shown it is intrinsically disordered with some minimal preferences for transient secondary structure and transient long-range contacts [31], [32], [33], [34], [35], [36], [37], [38]. p53TAD regulates the transcriptional activity and cellular stability of p53. This domain contains binding sites for the ubiquitin ligase, MDM2, the 70 kDa subunit of replication protein A, RPA70, and numerous kinases and phosphatases. When bound to MDM2, p53 becomes ubiquitinated and targeted for proteasome-mediated degradation. When bound to RPA70, p53 may be stabilized and available to amplify the cellular response to DNA damage. The transient secondary structure observed in free p53TAD is stabilized when bound to either protein [36], [39], [40].

**Figure 1:**
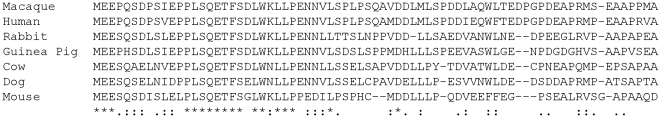
Protein sequence alignment of the p53 transactivation domain from seven mammals. The protein sequences correspond to residues 1–74 of human p53. Percent identity ranges from 91% (between human and macaque) to 42% (between dog and mouse).

Figure 1 shows protein sequence alignments for p53TAD from seven mammals. Sequence identity for these homologues ranges from 91% between human and macaque to 42% between dog and mouse. Previous work on ordered proteins has shown that amino acid sequence identity of greater than 40% leads to nearly identical protein folds that often have identical functions [41], [42], [43], [44], [45]. We are currently investigating whether there is a similar relationship between sequence and structure for the p53TAD homologues shown in Figure 1. As part of this investigation, NHNOE data was collected for human, dog, mouse, cow, guinea pig, and rabbit p53TAD and compared with disorder probabilities from VL-XT, IUPred, and VSL2B. Significant correlations were observed between the NHNOE and disorder probabilities, indicating that all three are accurate predictors of backbone dynamics at the level of single amino acid residues.

## Results and Discussion

### Correlating backbone dynamics and disorder probabilities of p53TAD homologues

Backbone resonance assignments and NHNOE values were obtained for the non-proline residues from human, dog, mouse, cow, guinea pig, and rabbit p53TAD (See materials and methods). The measured NHNOE values for each homologue were converted to NHNOE* values by taking the antilog and dividing this number by the maximum resulting value. This produces a number that varies between 0 and 1, with 0 corresponding to the most flexible residues and 1 corresponding to the least flexible. This number can be plotted on the same scale as the disorder probability plots, allowing a test of their residue specific accuracy.

**Figure 2:**
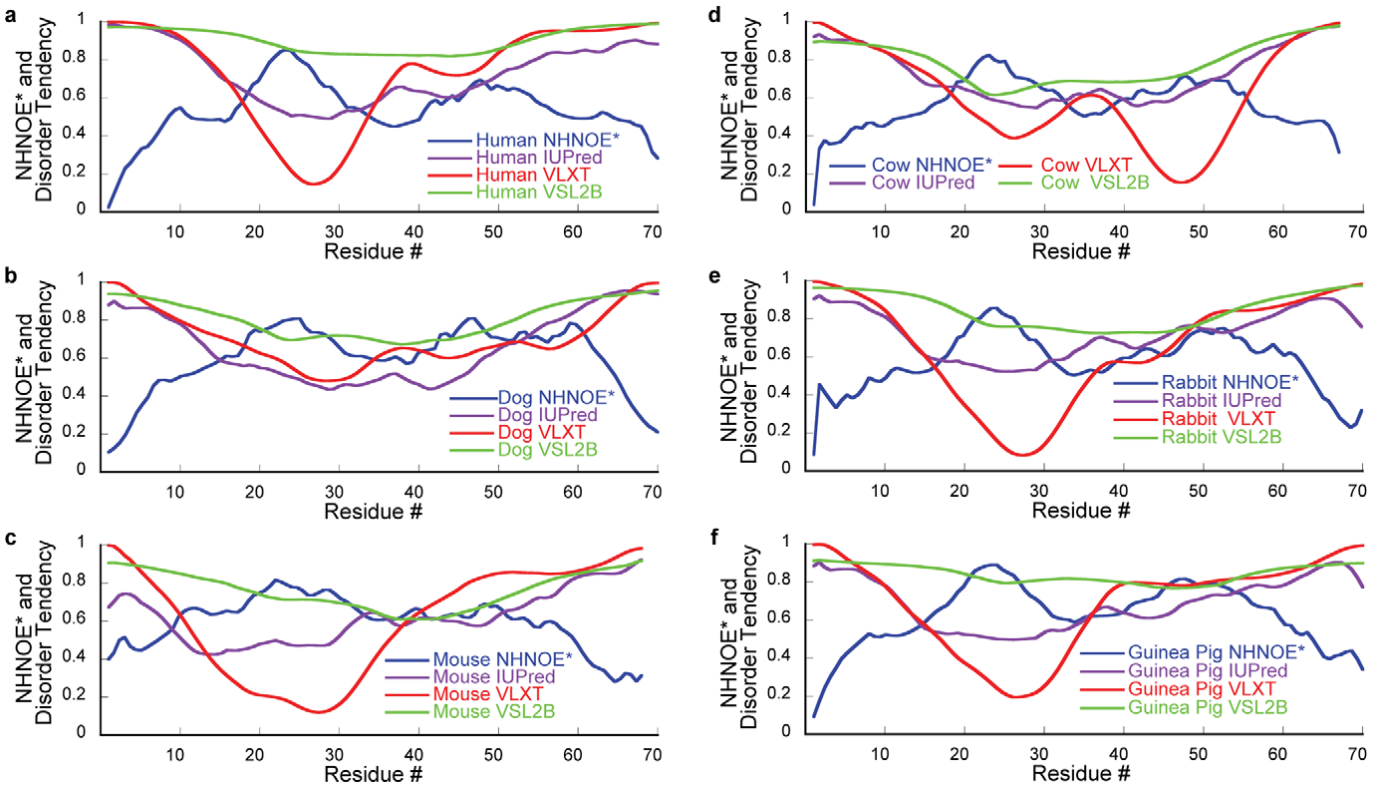
NHNOE* and Disorder probabilities smoothed over a 5 residue window. (a) Human, (b) Dog, (c) Mouse, (d) Cow, (e) Guinea Pig, and (f) Rabbit p53TAD.

Figure 2 shows plots of the NHNOE* values and the disorder probabilities calculated by VL-XT, IUPred, and VSL2B for residues 1–70 of human, 1–70 of dog, 1–68 of the aligning mouse residues, 1–67 of cow, 1–70 of guinea pig, and 1–70 of rabbit p53TAD. These residue ranges were chosen for non-human homologues because the human construct does not include a polyproline domain that separates the transactivation domain from the sequence specific DNA binding domain of p53. The plots for all three homologues clearly show a negative correlation between the NHNOE* values and the disorder probabilities.

Strong correlations are observed near the termini. Many IDPs exhibit a general polymer effect where terminal residues are more flexible than interior residues. This is indicated by small NHNOE* values at the termini. VL-XT is expected to perform well at the termini because part of the training set was terminal residues. This can explain the correlation at the N-termini but not the C-termini, because the VL-XT predictions were based on the sequences of the full-length p53 proteins. To test whether there was an effect on the predictions at the C-terminus, the VL-XT predictions were repeated using just the p53TAD sequences. This did not result in significant differences in the disorder probabilities at either terminus. The IUPred predictions were made using the setting for long disordered regions, which does not correct for terminal residues. However, differences in the IUPred predictions were observed at the termini when sequences of p53TAD or full-length p53 were used. These differences are due to the fact that IUPred calculates interaction energies over a 100 residue sequential neighborhood. The best correlations were observed between the NHNOE* values and the IUPred predictions using only the p53TAD sequences. A previous study comparing the ensemble structure of human p53TAD to the same domain in the full-length protein showed there is no interaction between p53TAD and the rest of p53, so it is reasonable to use the IUPred predictions for p53TAD [37]. Like VL-XT, the VSL2B training set included N- and C-terminal residues. Using VSL2B there are no significant differences at the C-termini in the disorder probabilities between the full length and p53TAD sequences. The PSI-BLAST feature set was excluded since it incorporates sequence homology, and this dataset compares homologues already.

Visual inspection of the plots in Figure 2 show a strong negative correlation between the residue specific backbone dynamics and the disorder probabilities of residues 15–30 for all three predictors and all six homologues. Residues 15–30 form the MDM2 binding region of p53. This region forms transient helical structure in the absence of MDM2 that is stabilized upon binding [34], [36], [40]. Formation of transient helical structure in IDPs increases the local rotational correlation time, corresponding to a reduction in overall molecular motion and a higher NHNOE*. The NHNOE* values for the six homologues show a peak in this region, which is consistent with the previous observations of transient helical structure for human p53TAD. Transient helical structure is also observed for residues 15–30 of the non-human p53TAD homologues (data not shown). All three predictors are sensitive to this behavior and show a dip (to some extent) in the MDM2 binding region. This behavior was expected for VL-XT. It was previously shown that this predictor is able to discriminate regions of IDPs that become ordered upon binding to a protein partner [46], [47]. The data presented in Figure 2 suggest that VL-XT, IUPred, and VSL2B are sensitive to regions of reduced flexibility in IDPs. These regions often correlate with protein binding regions and contain local elements of transient secondary structure. Although there is known transient secondary structure in these regions, VSL2B shows the smallest dips in the binding sites, which is surprising given its programming takes secondary structure prediction into account. However, the dips for VSL2B more accurately align with the NHNOE* peaks, whereas IUPred and VLXT are shifted toward the C-termini. Reasonable negative correlations between experiment and prediction were also observed for the RPA70 binding region, which encompasses residues 40–60 of human p53TAD. This region is not as conserved as the MDM2 binding region (see Figure 1) and is more dynamic. However, there are still small peaks for the NHNOE* values and small dips for all of the disorder probabilities in this region, with the exception of the VL-XT probabilities for mouse p53TAD.

### Regression Analysis

Linear regression was performed on the NHNOE* and disorder probabilities to assess the statistical significance of the correlations shown in Figure 2. Correlation coefficients and two-tailed p-values for the linear regression are shown in Table 1. All the p-values are less than 0.002 and in most cases are less than 0.0001, indicating the correlations are significant. While these three predictors were specifically designed to identify long disordered regions they can also accurately estimate the backbone dynamics of individual amino acids in these long disordered regions.

**Table 1:** Linear Regression of NHNOE* Versus Disorder Probability.

Species	Predictor	Sample Size	r	Two-tailed p-values
Human	IUPred	58	0.55	0.000008
Human	VL-XT	58	0.54	0.000012
Human	VSL2B	58	0.42	0.00103
Dog	IUPred	60	0.44	0.000435
Dog	VL-XT	60	0.65	<0.000001
Dog	VSL2B	60	0.49	0.000071
Mouse	IUPred	60	0.65	<0.000001
Mouse	VL-XT	60	0.58	0.000001
Mouse	VSL2B	60	0.51	0.000031
Cow	IUPred	59	0.59	0.000001
Cow	VL-XT	59	0.66	<0.000000
Cow	VSL2B	59	0.62	<0.000000
Guinea Pig	IUPred	61	0.58	0.000001
Guinea Pig	VL-XT	61	0.53	0.000014
Guinea Pig	VSL2B	61	0.71	<0.000000
Rabbit	IUPred	62	0.43	0.000524
Rabbit	VL-XT	62	0.43	0.000515
Rabbit	VSL2B	62	0.48	0.000065

**Figure 3:**
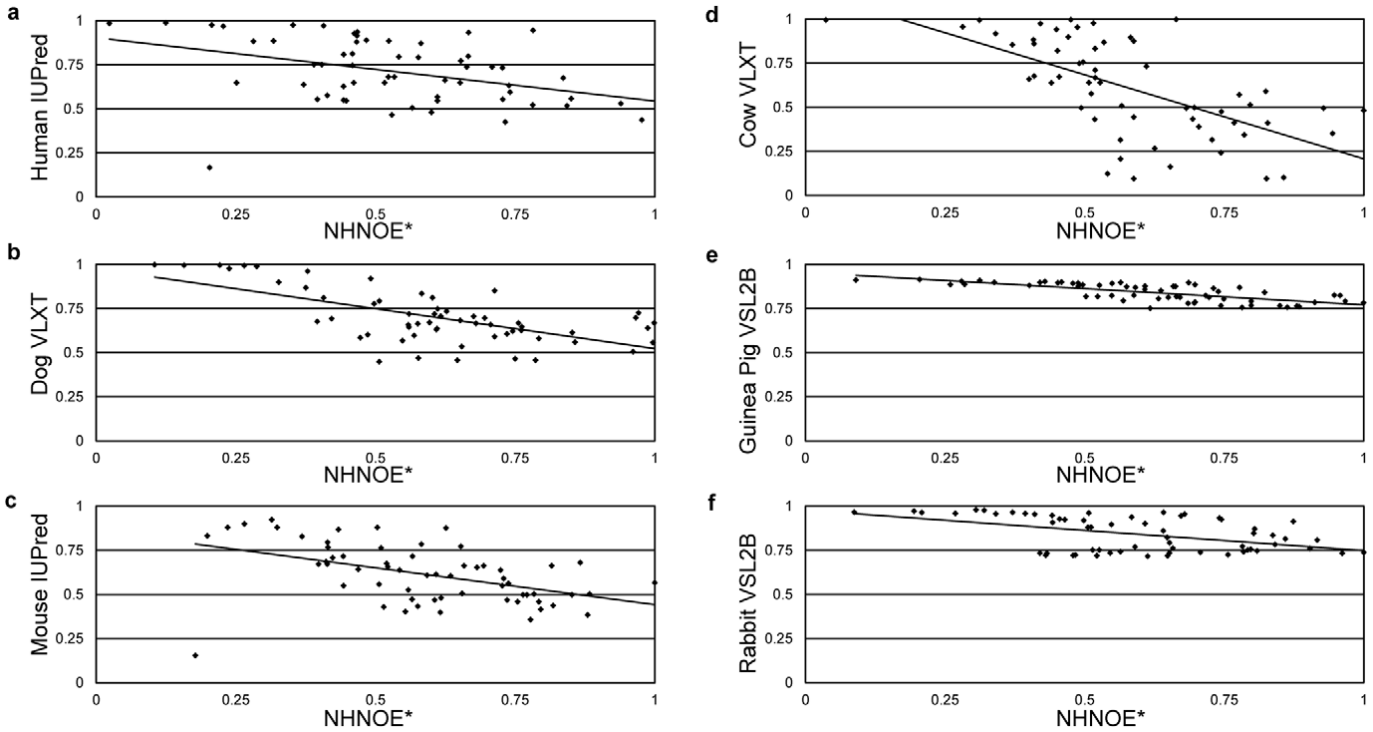
Correlation Plots of the Best fitting plots for Human IUPred. (a), Dog VL-XT (b), Mouse IUPred (c), Cow VL-XT (d), Guinea Pig VSL2b (e), and Rabbit VSL2b (f).

Figure 3 shows the correlation plot between NHNOE* and the disorder probabilities with the strongest correlation for each of the six homologues, respectively. There is no single predictor with the strongest correlation for a majority of the homologues; in fact the predictors split the homologues evenly, with each having the strongest correlation for two of the six homologues. Interestingly, the homologues appear to be apportioned according to their molecular flexibility. Based on the raw NHNOE data the overall molecular flexibility of the six homologues ranks in increasing disorder as follows: Guinea Pig<Rabbit<Mouse<Human< Cow<Dog. VLXT probabilities have the highest correlation with the two most dynamic homologues, dog and cow. VSL2B probabilities have the highest correlation with the two least dynamic homologues rabbit and guinea pig. IUPred probabilities have the highest correlation with the two homologues displaying intermediate dynamics, human and mouse. While this result is interesting, the dataset is too small to assign any statistical significance to this partitioning.

### Accuracy of residue specific correlations between backbone dynamics and disorder probability

**Figure 4:**
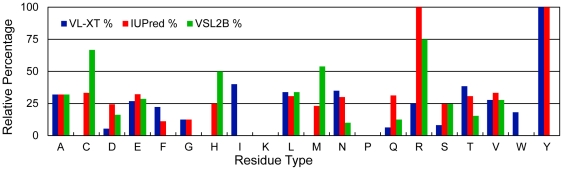
Relative percentage of incorrect predictions for VL-XT, IUPred, and VSL2B. An incorrect prediction is defined as a VL-XT, IUPred, or VSL2B value that is at least 1≥σ from the mean value estimated using the linear regression equations.

Figure 4 shows the relative percentages for amino acid residues with VL-XT, IUPred, or VSL2B values that were ≥1σ from the mean value estimated using the linear regression equations. Relative percentages were determined by identifying the number of times a particular residue type had a VL-XT, VSL2B or IUPred value that was ≥1σ from the mean. This number was then divided by the total number of a given residue type in the six sequences and converted to a percentage. All disorder probabilities from each of the three predictors were within 2σ of the linear regression means, which is a common cutoff used to identify outliers. A cutoff of 1σ was chosen for the current analysis because values of disorder predictors that are ≥1σ from the mean are structurally significant. For instance, W53 in human p53TAD has a VL-XT disorder probability of 0.94, which is ≥1σ but ≤2σ from the mean, this disorder probability corresponds to an NHNOE* value of 0.27, compared with the measured NHNOE* value of 0.66. The difference in the predicted and measured NHNOE* values for W53 is structurally significant and corresponds to the difference between an amino acid residue that is completely disordered versus one that has some transient secondary structure.

In Figure 4, relative percentages are zero for P because the NHNOE cannot be measured due to its lack of an amide hydrogen. Relative percentages are also zero for all K residues, but in this case all three predictors were within 1σ of the mean for these residues. The predictors make an average of 22.0%±3.98% errors, with disorder probabilities ≥1σ from the mean, excluding residues that do not occur at least once per homologue. Only the A, E, L, and V residues are above this range for all three predictors. This indicates that VL-XT, VSL2B, and IUPred are inaccurately predicting the dynamic behavior for these residue types. Probability values ≥1σ for A and E residues occur most frequently above the regression line, which indicates that all three algorithms predict these residue types to be less ordered than they actually are. For A residues there are 17 predictions that are greater than the mean and 6 that are less, while for E residues there are 29 predictions that are greater than the mean and 21 that are less. Conversely L and V probabilities ≥1σ from the mean are most often below the regression line, which indicates that all three algorithms predict these residue types to be more ordered than they actually are. For L residues there are 47 predictions that are less than the mean and 15 that are greater, while for V residues there are 10 predictions less than the mean and 7 that are greater.

L, and V were previously classified as order-promoting residues based on their low frequency of occurrence in a database of experimentally characterized disordered proteins [48]. A was classified as a neutral amino acid, while E was classified as a disorder promoting residue. The frequency of L and E residues in the p53TAD homologues are approximately 90% and 70% higher than the values observed in the database, respectively. A and V residues are approximately 20% and 30% less frequent in the p53TAD homologues than in the database, respectively. According to the NHNOE, L and V residues are more dynamic than predicted, while A and E residues are less dynamic than predicted. L is particularly interesting because over 75% of disorder probabilities ≥1σ from the mean value are predicted to be more ordered than the dynamics data indicate. The high frequency of an order-promoting residue may account for why VL-XT, VSL2B, and IUPred make so many incorrect predictions for L residues in these p53TAD sequences.

### Summary

In this report, we show that three popular algorithms for predicting disorder probability based on amino acid sequence can accurately estimate the backbone dynamics of individual amino acids in long disordered regions from a family of disordered proteins. These findings are consistent with the results from three previous studies, which observed correlations between backbone dynamics and disorder probability using either molecular dynamics simulations or NMR dynamics measurements. In one of these studies, increased internal flexibility was suggested by both disorder predictors and molecular dynamics simulations [49]. In a second study, the insertion of a β-hairpin between two chimeric proteins resulted in a significant increase in predicted disorder probabilities that were subsequently confirmed using NMR dynamics measurements [50]. The third study found a strong negative correlation between the degree of predicted disorder and the stability of the protein complexes. In the third study, molecular dynamics simulations were used to show that binding regions with higher predicted disorder probabilities correlated with weaker complex formation [51]. While the dataset used for this comparison is small, the correlations observed are robust, and demonstrates the accuracy of the disorder predictors for backbone dynamics at single amino acid resolution. As more experimental data on the backbone dynamics of IDPs is collected we predict this relationship will be refined so that in the future the NHNOE and other NMR measurements that provide information about residue specific structure and dynamics can be used to guide the development of disorder predictors.

## Materials and Methods

### Protein purification

Samples of human p53TAD (residues 1–73) that were uniformly labeled with either ^15^N or ^15^N and ^13^C, were prepared as previously described [36]. Samples for dog (residues 1–77), mouse (residues 1–87), cow (residues 1–82), guinea pig (residues 1–88), and rabbit (residues 1–87) p53TAD were prepared using this same method.

### NMR data collection and analysis

Resonance assignments for human p53TAD were previously reported [36]. Experiments on mouse, dog, cow, guinea pig, and rabbit p53TAD were carried out at 25°C on a Varian VNMRS 600 MHz spectrometer equipped with a triple resonance pulse field *Z*-axis gradient cold probe. To make the amide ^1^H and ^15^N as well as ^13^C_α_, ^13^C_β_ and ^13^CO resonance assignments, sensitivity enhanced ^1^H-^15^N HSQC and three dimensional HNCACB and HNCO experiments were performed on the uniformly ^15^N and ^13^C labeled samples of dog (0.47 mM), mouse (0.36 mM), cow (0.38 mM), guinea pig (0.57 mM), and rabbit (0.341 mM) p53TAD in 90%H2O/10% D2O, PBS buffer, at a pH of 6.8. For the HNCACB experiment, data were acquired in ^1^H, ^13^C and ^15^N dimensions using 8012.8 (*t*_3_)×12000 (*t*_2_)×2000 (*t*_1_) Hz sweep widths, and 512 (*t*_3_)×128 (*t*_2_)×32 (*t*_1_) complex data points. For the HNCO, the sweep widths were 8012.8 (*t*_3_)×3770 (*t*_2_)×2000 (*t*_1_) Hz, complex data points were identical to the HNCACB. The sweep widths and complex points of the HSQC were 8012.8 (*t*_2_)×2000 (*t*_1_) Hz and 512 (*t*_2_)×128 (*t*_1_), respectively. For dog p53TAD, processing and analysis of the HNCACB data resulted in 65 non-proline, amide ^1^H, ^15^N, ^13^C_α_ and ^13^Cb resonance assignments plus 10 proline ^13^C_α_ and ^13^C_β_ resonance assignments. 63 ^13^CO resonance assignments were gained from HNCO data analysis. For mouse p53TAD, 75 non-proline, amide ^1^H, ^15^N, ^13^C_α_ and ^13^C_β_ resonance assignments plus 12 proline ^13^C_α_ and ^13^C_β_ resonance assignments were gained from HNCACB. 72 ^13^CO resonance assignments were gained from HNCO data analysis. For cow p53TAD, processing and analysis of the HNCACB data resulted in 67 non-proline, amide ^1^H, ^15^N, ^13^C_α_ and ^13^C_β_ resonance assignments plus 12 proline ^13^C_α_ and ^13^C_β_ resonance assignments. 65 ^13^CO resonance assignments were gained from HNCO data analysis. For rabbit p53TAD, processing and analysis of the HNCACB data resulted in 71 non-proline, amide ^1^H, ^15^N, ^13^C_α_ and ^13^C_β_ resonance assignments plus 12 proline ^13^C_α_ and ^13^C_β_ resonance assignments. 71 ^13^CO resonance assignments were gained from HNCO data analysis. For guinea pig p53TAD, processing and analysis of the HNCACB data resulted in 73 non-proline, amide ^1^H, ^15^N, ^13^C_α_ and ^13^C_β_ resonance assignments plus 11 proline ^13^C_α_ and ^13^C_β_ resonance assignments. 74 ^13^CO resonance assignments were gained from HNCO data analysis

All NMR spectra were processed with nmrPipe and analyzed using nmrView software [36], [52]. Apodization was achieved in the ^1^H, ^13^C and ^15^N dimensions using a squared sine bell function shifted by 70°. Apodization was followed by zero filling to twice the number of real data points and linear prediction was used in the ^15^N dimension of the HNCACB and HNCO. The ^1^H carrier frequency was set on the water peak, and 4.753 ppm was used as the reference frequency in this report.

^1^H-^15^N steady-state NOE experiments were recorded in the presence and absence of a 120° off-resonance ^1^H saturation pulse every 5 ms for a total of 3 s. A total of 512 (*t*_2_)×128 (*t*_1_) complex points were recorded with 128 scans per increment. The NHNOE values were determined by taking the quotient of the intensity for resolved resonances in the presence and absence of proton saturation. Three measurements were made on each protein and the values were averaged.

### Disorder Prediction

For IUPred (iupred.enzim.hu) the N-terminal residues comprising the TAD were input using the long disordered region prediction setting, human 1–90, dog 1–77, mouse 1–87, cow 1–82, rabbit 1–87, and guinea pig 1–88. The VL-XT (www.pondr.com) predictions represent the full length predictions for each homologue. For the VSL2B (ist.temple.edu) predictions the full protein sequences were entered, using only the VSL2B feature set.
